# Views and experiences of healthcare professionals towards the use of African traditional, complementary and alternative medicines among patients with HIV infection: the case of eThekwini health district, South Africa

**DOI:** 10.1186/s12906-015-0687-3

**Published:** 2015-06-06

**Authors:** Manimbulu Nlooto

**Affiliations:** Discipline of Pharmaceutical Sciences, School of Health Sciences, University of KwaZulu-Natal, Westville Campus, Durban, South Africa

**Keywords:** HIV management, Complementary, Alternative, Traditional medicines, Healthcare providers, Communication

## Abstract

**Background:**

Many patients with human immunodeficiency virus infection use traditional, complementary, and alternative medicines and other practices to combat the disease, with some also using prescribed antiretroviral therapy provided by the public health sector. This study aimed to establish the awareness of public sector biomedical health care providers on the use of traditional, complementary and alternative medicines by HIV-infected patients who also used highly active antiretroviral therapy, and to determine whether this was based on patients seen or cases being reported to them. Potential risks of interactions between the prescribed antiretroviral and non-prescribed medication therapies may pose safety and effectiveness issues in patients using both types of treatment.

**Methods:**

A descriptive cross-sectional study, using a researcher administered semi-structured questionnaire, was conducted from June to August 2013 at ten public sector antiretroviral clinics in five regional, three specialised and two district hospitals in eThekwini Health District, South Africa. Questionnaires were administered through face-to face interview to 120 eligible participants consisting of doctors, nurses, pharmacists and post-basic pharmacist assistants in HIV clinical practice. The results are presented as percent or proportion with standard error (SE), or as frequency.

**Results:**

Ninety-four respondents completed the questionnaire, yielding a response rate of 78.3 %. Almost half (48/94) were aware of patients using African traditional herbal medicines, over-the-counter supplements, unnamed complementary Ayurveda medicines and acupuncture. Twenty-three of the 94 respondents (24.4 %) said they had consulted patients who were using both antiretroviral therapy and certain types of non-prescribed medication in the previous three months.

**Conclusions:**

Awareness among healthcare providers on patient use of traditional, complementary and alternative medicines was relatively high. Few respondents had seen patients who used mostly African traditional medicines, over-the counter supplements, and negligible complementary Ayurveda medicines and acupuncture, with caution being advised in the interpretation of the former. Further research is needed to investigate communication between healthcare providers and patients in this regard, and levels of acceptance of traditional, complementary and alternative medicines by biomedical health care workers in HIV public sector practice.

## Background

While many patients in South Africa with human immunodeficiency virus (HIV) infection use traditional, complementary and alternative medicines, there is a scarcity of research among public sector biomedical health care providers on their awareness of this practice, and on the level of disclosure by patients. This study aimed to establish the awareness of healthcare providers about their HIV-infected patients’ use of traditional, complementary and alternative medicines (TCAM) besides other prescribed medication, and to determine whether this was based on patients seen or cases being reported to them. Access to highly active antiretroviral therapy (HAART) by all those needing treatment is one of the goals to fight the HIV pandemic in South Africa. At of the end of 2012, two million people were estimated to have access to HAART in the country [1]. With the widespread use of HAART, HIV has been transformed into a complex chronic disease, with patients living longer. While the benefits of antiretroviral therapy (ART) are well established, the development of toxicity is one of the limitations to treatment success [2]. In addition to ART, many HIV infected patients also use complementary and alternative medicines to cope with the disease [3], the extent of which is unknown.

Use of herbal therapies in KwaZulu-Natal Province (KZN) was estimated in 2008 to be practiced by 36.6 % of HIV-infected persons prior to starting HAART, at 8.0 % after six months, at 4.1 % after 12 months and 0.6 % after 20 months post initiation [4]. The use of non-prescribed medicines by HIV-infected patients was estimated at 15.6 % in patients on ART in Pretoria, South Africa [5]. In Uganda, patients on HAART for less than four years, and those who experienced antiretroviral related side-effects, were reported to be more likely to use traditional herbal medicines [6].

According to the World Health Organization, Traditional Medicines include *“diverse health practices, approaches, knowledge and beliefs incorporating plant, animal and/or mineral based medicines, spiritual therapies, manual techniques and exercises, applied singularly or in combination to maintain well-being, as well as to treat, diagnose or prevent illness* [7]*.”* The organization further defines complementary and alternative medicines as *“*a*broad set of health care practices that are not part of a country’s own tradition, or not integrated into its dominant health care systems* [8]*.”*

However, the lack of disclosure of the use of TCAM to health care providers is a cause of concern, with almost one half of HIV-infected women in the United States using any type of such medication not having discussed its use to their biomedical health care providers [9]. A study at two sites in South Africa reported 15.5 % (15/97) of HIV-infected patients using traditional medicine and antiretroviral therapy, the majority of whom (92.9 %) chose not to disclose their use to the public sector biomedical health care providers [10]. Building a doctor-patient relationship, opening health care discussions, and obtaining information are amongst the healthcare providers responsibilities towards their patients [11]. Communication between health care providers and patients plays a key role in addressing adverse events [12].

Patients may use TCAM for various reasons, such as to strengthen immunity or improve general well-being, as well as in cases where prescribed medication does not work [13]. However, the potential for direct physical harm is a real challenge, with contra-indications and the negative interactions between some prescribed ART medicines and natural products being described [14]. In the private and public health sectors, doctors play an important role in managing HIV infected patients through partnerships with them, and assistance is needed from nurses, pharmacists, social workers, dieticians and support groups to ensure a holistic management of the patient [15]. In South Africa, the multidisciplinary team caring for HIV-infected patients in the public health sector consists of doctors, nurses, pharmacists and post-basic pharmacist assistants, amongst other health care workers. As part of this holistic management, one of the challenges facing the health care providers is ensuring the safety of ART, which requires full disclosure of prescribed medication and other non-prescribed medication therapies used by HIV-infected patients. Challenges brought about by using TCAM in combination with allopathic ART calls for accurate information to be provided by the patient [16].

Many studies have reported on the self-reported use of TCAM by patients, while this study explored the views and experiences of the biomedical healthcare providers. No contact was made with patients and traditional healers who are also healthcare providers. Building on previous work conducted in the eThekwini Health District [15, 16], this study reports on the awareness of public health sector biomedical healthcare providers about patient’s disclosure of the use of TCAM besides other prescribed medication. This was done in cases seen by four categories of healthcare providers among HIV-infected patients attending public health sector antiretroviral clinics.

## Methods

### Study design, participants and sites

A descriptive cross-sectional study using a researcher administered semi-structured questionnaire was conducted from June to August 2013 at ten public accredited antiretroviral clinics within five regional, three specialised and two district hospitals in eThekwini Health district, South Africa. The provincial list of public accredited antiretroviral sites contained 16 locations in the eThekwini Health District at the time of data collection, with this study being conducted in the ten sites where permission was obtained from the relevant gate keepers.

Questionnaires were administered through face-to face interview during clinic visit hours to 120 eligible participants, namely: doctors, nurses, pharmacists and post basic pharmacist assistants (PBPA) in HIV clinical practice, who had a minimum of three months work experience in HIV clinical practice. To minimise sampling bias, assuming an expected 10 % prevalence of awareness on the use of TCAM among respondents within a ±5 % precision, an estimated sample size of 138 was calculated following a formula described in the literature [17]. The questionnaire was pilot tested prior to data collection, and those participants who agreed to participate were requested to sign consent before being interviewed. This study enrolled 120 eligible participants due to a scarcity of these four categories of biomedical healthcare providers at public antiretroviral sites. Respondents who did not answer all the questions, due to their unavailability for leave or meeting attendance within the study time frame, were excluded from the final analysis. A group of nine final year pharmacy students administered the questionnaire, which consisted of questions on the following five categories: (i) socio-demographic characteristics; (ii) awareness about disclosure of prescribed treatments besides ART and non-prescribed TCAM; (iii) biomedical healthcare providers’ reported cases with HIV-infected patients using both ART and TCAM; (iv) reasons for uses and non-disclosure of information about TCAM by HIV-infected patients; (v) and other pharmacovigilance related information. This was done to establish possible interactions between antiretroviral medicines and use of TCAM, as described in the South African standard treatment guidelines [18].

### Statistical analysis

All collected data was entered into Excel and later analysed using the SPSS statistical programme for Windows, version 20. The results are presented as percent or proportion with standard error (SE), and relative error (RE). If the relative standard error was0.50 or greater, the percent was not stable and could not be reported when the number of observed events in a given table cell is less than five. Mean with standard deviation was reported for continuous variables, while categorical variables were described as frequency, with bar graph representation where applicable.

### Ethics statement

This study received ethical approval under reference number SHSEC 030/13 from the School of Health Sciences, University of KwaZulu-Natal. Permission was sought from Department of Health and hospital management gatekeepers before entering the premises of all surveyed health facilities. Participation in the study was voluntary, and an information sheet form was given to respondents who signed informed consent. All questionnaires were coded to ensure full anonymity of respondents and sites.

## Results

Ninety-four respondents completed the questionnaire within the study time frame, yielding a response rate of 78.3 %.

### Socio-demographic characteristics

There were 25 doctors (26.6 %,), 29 nurses (30.9 %), 32 pharmacists (34.1 %) and eight post-basic pharmacist assistants (8.5 %). The majority of respondents were female (67/94) and had up to five years of work experience (53/94). The overall mean professional experience was 5.9 years (standard deviation ±4.4, range 0.4 to 22.0 years). Table 1 presents the socio-demographic characteristics of the respondents.

**Table 1:** Socio-demographic characteristics of the health professional respondents (n = 94)

Category	Sub-category	Number, (%), SE, RE
Gender	Female	67 (71.3 %), SE (0.055), RE (0.0008)
	Male	27 (28.7 %), SE (0.087), RE (0.003)
Professional qualification	Doctor	25 (26.6 %), SE (0.088),RE (0.033)
	Nurse	29 (30.9 %),SE (0.086),RE (0.0027)
	Pharmacist	32 (34.0 %), SE (0.084),RE (0.0024)
	PBPA	8 (8.5 %), SE (0.099),RE (0.011)
Work experience(years)	0.3–5	53 (56.4 %),SE (0.068),RE (0.0012)
	6–25	41 (43.6 %),SE (0.077),RE (0.0017)
	Mean (standard deviation)	5.9 (±4.4)
	Range (minimum -maximum)	0.3–22.0

Legend: SE = standard error, RE = relative error, PBPA = Post basic pharmacist assistant

### Awareness about disclosure of prescribed treatments besides ART and non-prescribed TCAM

Of the 94 respondents, 40 (42.5 %, SE0.078, RE0.001) reported that HIV-infected patients disclosed the use of other prescribed medication besides ART. Almost half of the respondents agreed that they were aware of patients using African traditional herbal medicines, over-the-counter supplements, unnamed complementary Ayurveda medicines and acupuncture. In addition, 23 participants (24.4 %) stated that they were familiar with such practices, and had cases with patients using both antiretroviral therapy and a few types of TCAM in the three months prior to data collection. Table 2 indicates the number of doctors, nurses, pharmacists and post basic pharmacist assistants who reported being aware of the use by HIV patients of other prescribed medication, besides ART, as well as TACM medicines.

**Table 2:** Healthcare workers’ awareness of the use of prescribed and non-prescribed medications by HIV patients

Respondent category (No)		Aware of patient disclosure of all other prescribed medication	Aware of patient use of TCAM	Cases with patients using both ART and TCAM
Doctors (n = 25)	Yes	7 (7.45 %), SE (0.052), RE (0.007)	18 (19.15 %), SE (0.087), RE (0.0045)	7 (7.45 %), SE (0.052), RE (0.007)
	No	18 (19.15 %)	7 (7.45 %)	18 (18.08 %)
Nurses (n = 29)	Yes	15 (15.96 %), SE (.0.068), RE (0.004)	19 (20.21 %), SE (0.075), RE (0.0037)	6 (6.38 %), SE (0.046), RE (0.0072)
	No	14 (14.90 %)	10 (10.64 %)	23 (24.47 %)
Pharmacists (n = 32)	Yes	15 (15.96 %), SE (0.065), RE (0.004)	9 (9.45), SE (0.052), RE (0.005)	7 (7.45 %), SE (0.052), RE (0.007)
	No	18 (19.15 %)	23 (24.47 %)	25 (26.60 %)
PB PA (n = 8)	Yes	3 (3.19 %), SE (0.165), RE (0.052)	2 (2.13 %), SE (0.051), RE (0.024)	3 (3.19 %), SE (0.062), RE (0.019)
	No	5 (5.32 %)	6 (6.38)	5 (5.32 %)
Total (n = 94)	Yes	40	48	23
	No	54	46	71

Legend: PBPA = post basic pharmacist assistant, TCAM = traditional, complementary and alternative medicines

Note: standard error calculated on the proportion of respondents who said yes to the question

**Fig. 1:**
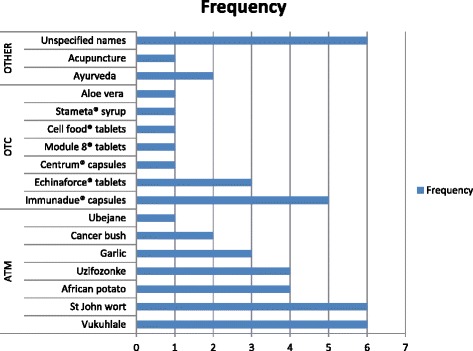
Types of non-prescribed medication. Legend: ATM = African traditional herbal medicines, OTC = over-the-counter

Figure 1 illustrates the types of TCAM (48/94) listed by respondents as being used by HIV patients. They consisted of certain African traditional herbal medicines, over-the counter supplements, Ayurveda medicines and acupuncture.

### Biomedical healthcare providers’ reported cases with HIV-infected patients using both ART and TCAM

Reported cases with patients who used both ART and TCAM (23/94) consisted of the following medication therapies: (i) Echinaforce® tablets (3); (ii) unnamed African herbal mixtures (3); (iii) garlic (2); (iv) cancer bush (2); (v) Vukuhlale (2); (vi) Stameta® (2); (vii) African potato (2); (viii) immunadue® (2); (ix) over-the-counter centrum tablets (2); (x) unnamed Ayurveda preparations (2); and (xi) acupuncture (1).

### Reasons for uses and non-disclosure of information about TCAM by HIV-infected patients

**Fig. 2:**
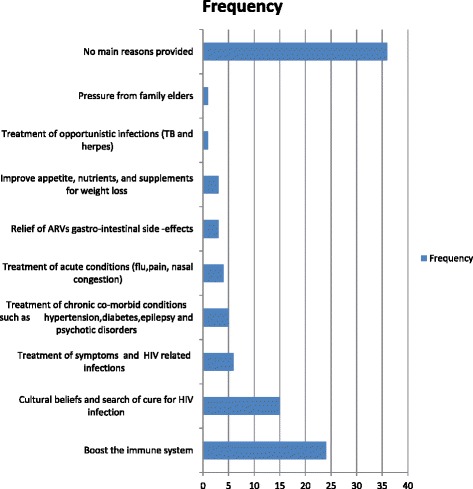
Perceived main reasons reported by healthcare providers for using non-prescribed medication by patients. Legend: ARV = Antiretroviral medicine, TB = tuberculosis, HIV = human immunodeficiency virus

Healthcare providers were asked to provide one perceived main reason why they thought patients resorted to using TCAM, with the results being presented in Fig. 2. The respondents indicated that they thought patients reasons were mainly: boost their immune system (7/23), cultural beliefs of beneficial effects of traditional herbal mixtures (6/23), treatment of gastro-intestinal side-effects, pain and chronic illness (5/23), nutrients (2/23), relief of disease symptoms and improve survival (2/23), and pressure from family members (1/23).

**Fig. 3:**
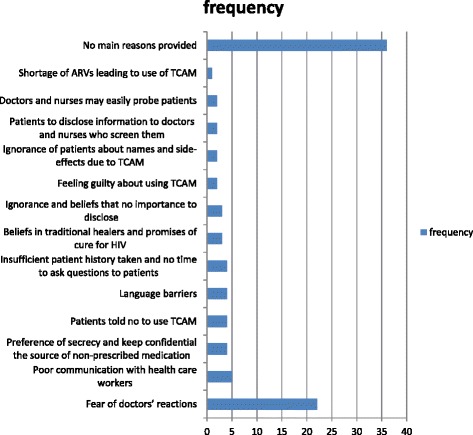
Perceived main reasons reported by healthcare providers for non-disclosure of the use of TCAM by HIV-infected patients. Legend: TCAM = traditional, complementary and alternative medicines

Respondents indicated the main reasons they thought for non-disclosure of information about the use of TCAM by HIV patients to biomedical healthcare providers, as illustrated in Fig. 3. Seven of the 94 respondents (7.45 %) reported the use of TCAM practices as perceived causes of adverse events. While very few doctors (1/94) and pharmacists (1/94) perceived the use of TCAM as main causes of adverse events, none of the nurses and post basic pharmacist assistants listed the use of TCAM as main causes of adverse events. One nurse (1/94) viewed TCAM as alternate causes of adverse events, while one doctor (1/94) and one nurse (1/94) listed OTC supplements as main causes of adverse events. None of the 23 respondents who had seen patients or heard about them in their practice had also associated the use of TCAM with adverse events and probable interactions with prescribed antiretroviral medicines.

## Discussion

Disclosure prevalence of the use of complementary and alternative medicine among HIV-infected women was estimated at 36 % in both pre- and post-HAART eras in the United States [9]. Our findings, of few respondents (24.4 %) having seen patients, or hearing about the use of TCAM and prescribed ART, may be due to the decline of self-reported use of herbal medicines by HIV-infected patients following ARV initiation, as reported in a cohort of HIV-infected patients in Kwazulu-Natal [4]. However, a relatively high rate of awareness among healthcare providers about the use of African traditional herbal medicines by HIV patients was found when compared to another study in Uganda [19].

Regarding the reasons for use and non-disclosure, poor communication between doctors and HIV-infected patients was identified as a contributor to low rates of disclosure of the use of traditional, complementary and traditional medicines by patients to healthcare providers in Uganda [19]. Non-disclosure of the use of herbal medicines to medical doctors was estimated at 92.3 % among HIV patients, who also reported never being asked by their physician about their use of herbal medicines in western Uganda [20]. The lack of proper communication between patients and health care workers is a real challenge, aggravated by negative perceptions and attitudes of biomedical health care providers towards traditional methods of care [21].

A holistic approach to managing HIV-infected patients was discussed in a focus group with private sector doctors as one of the factors to promote adherence to antiretroviral therapy in the eThekwini Health District, KwaZulu-Natal [15]. This included the need for doctors and other health care workers to encourage the appropriate use of traditional medicine, provided that continuity of antiretroviral therapy and potential drug-herb interaction monitoring was assured. A greater awareness about the use of traditional, complementary and alternative medicines among healthcare workers requires more accurate information on what happens in real-life with regard to such practices. While a study showed that biomedical health care providers in Durban antiretroviral clinics admitted the personal use of traditional, complementary, and alternative medicines for various purposes [16], this does not guarantee the safety and effectiveness of such practices in patients on antiretroviral therapy. In Trinidad, an assessment of the acceptance of herbal medicines by physicians revealed that 40.6 % had used this treatment modality [22]. In Alabama and Georgia (USA) non-adherence to antiretroviral therapy was 1.69 times higher in HIV-positive women who used complementary and alternative medicines than among non-users of such practices [23]. Other potential health risks of complementary alternative medicines in HIV patients were discussed in a study in UK, where, besides adherence to antiretroviral therapy, serious drug interactions with antiretroviral therapy or adverse effects of the remedy used were reported with Echinacea, garlic, St John’s wort and aloe vera [24].

Pharmacovigilance activities among health care workers need to be informed by empirical evidence, based on people’s activities and sound research. Although perceived causality of adverse events due to the use of traditional, complementary and alternative medicines was very low in this study, suggesting the difficulty of attributing them to traditional, complementary and alternative medicines; a study in Zimbabwe reported abdominal pain and rash in association with the use of herbal remedies [25]. Occurrence of at least one or more adverse events related to use/misuse of over-the-counter medication was also reported among HIV-infected patients on antiretroviral therapy in Texas, USA [26]. Further traditional, complementary and alternative medicine education for health care workers is an important part of public health promotion in poor constrained settings, as has been discussed in developed world [27].

### Strengths, limitations and generalizability of the study

Although we could not collect the expected response rate of ≥ 80 %, a good response rate of 78.3 % was achieved, compared to the 60 % being the goal for most research of this type [28]. Missing information from those participants who could not provide answers to all questions within the study time frame was also a limitation. A 100 % response rate may have revealed other types of African traditional herbal medicines and other practices used by patients in conjunction with prescribed antiretroviral therapy. Views and experiences of other healthcare providers such as social workers, psychologists, dieticians and lay counsellors were not assessed despite their being part of a multidisciplinary team caring for HIV patients. No attempt was made to access patients’ medical records, which may have provided other findings in patients seen or cases being reported to healthcare providers. A larger and more inclusive sample may have provided more information on awareness about the use of traditional, complementary and alternative medicines among healthcare workers. The results of this study cannot be assumed to accurately reflect the TCAM practices of patients, and additional studies are needed that obtain information from them in this regard. Findings of this study cannot be generalised to the entire HIV clinical practice.

## Conclusions

Awareness among the biomedical healthcare providers about the use of TCAM by HIV patients was relatively high in the 10 public sector HIV clinics in eThekwini Health District. However, only a few respondents had seen patients who used them, most respondents indicating that they heard about such practices. The types of TCAM used were mainly African traditional medicines and over-the-counter supplements, while the use of complementary Ayurveda medicines and acupuncture practices was reported to be negligible. Regarding the potential harm of natural products contributing to adverse events and potential interactions with prescribed antiretroviral medicines, caution is advised in the interpretation of our findings due to the few reported cases of patients on using African traditional herbal medicines. Further research is needed to investigate communication between healthcare providers and patients in this regard, and attitudes towards the use of traditional, complementary and alternative medicines by biomedical healthcare providers in HIV public sector practice for a more holistic approach to HIV management.

## Abbreviations

HIV: Human immunodeficiency virus
ART: Antiretroviral therapy
HAART: Highly active antiretroviral therapy
PBPA: Post basic pharmacist assistant
SE: Standard error
RE: Relative error
TCAM: Traditional, complementary and alternative medicines
ATM: African traditional herbal medicines
OTC: Over-the-counter supplements
ARV: Antiretroviral medicine
